# Early assessment of tumor response to photodynamic therapy using combined diffuse optical and diffuse correlation spectroscopy to predict treatment outcome

**DOI:** 10.18632/oncotarget.15720

**Published:** 2017-02-25

**Authors:** Patricia Thong, Kijoon Lee, Hui-Jin Toh, Jing Dong, Chuan-Sia Tee, Kar-Perng Low, Pui-Haan Chang, Ramaswamy Bhuvaneswari, Ngian-Chye Tan, Khee-Chee Soo

**Affiliations:** ^1^ Division of Medical Sciences, National Cancer Centre, Singapore; ^2^ Division of Bioengineering, School of Chemical and Biomedical Engineering, Nanyang Technological University, Singapore; ^3^ Division of Surgical Oncology, National Cancer Centre, Singapore; ^4^ Nanyang Technological University, Singapore; ^5^ Harvard Medical School and Wellman Center for Photomedicine, Massachusetts General Hospital, Boston, MA, USA; ^6^ Daegu Gyeongbuk Institute of Science and Technology, Daegu, Korea

**Keywords:** photodynamic therapy, treatment response monitoring, optical spectroscopy, tissue oxygenation, relative blood flow

## Abstract

Photodynamic therapy (PDT) of cancer involves the use of a photosensitizer that can be light-activated to eradicate tumors via direct cytotoxicity, damage to tumor vasculature and stimulating the body's immune system. Treatment outcome may vary between individuals even under the same regime; therefore a non-invasive tumor response monitoring system will be useful for personalization of the treatment protocol. We present the combined use of diffuse optical spectroscopy (DOS) and diffuse correlation spectroscopy (DCS) to provide early assessment of tumor response. The relative tissue oxygen saturation (rStO2) and relative blood flow (rBF) in tumors were measured using DOS and DCS respectively before and after PDT with reference to baseline values in a mouse model. In complete responders, PDT-induced decreases in both rStO2 and rBF levels were observed at 3 h post-PDT and the rBF remained low until 48 h post-PDT. Recovery of these parameters to baseline values was observed around 2 weeks after PDT. In partial responders, the rStO2 and rBF levels also decreased at 3 h post PDT, however the rBF values returned toward baseline values earlier at 24 h post-PDT. In contrast, the rStO2 and rBF readings in control tumors showed fluctuations above the baseline values within the first 48 h. Therefore tumor response can be predicted at 3 to 48 h post-PDT. Recovery or sustained decreases in the rBF at 48 h post-PDT corresponded to long-term tumor control. Diffuse optical measurements can thus facilitate early assessment of tumor response. This approach can enable physicians to personalize PDT treatment regimens for best outcomes.

## INTRODUCTION

### Fluorescence imaging and photodynamic therapy

Photodynamic therapy (PDT) is an emerging cancer treatment modality that involves the use of a light-activatable photosensitizer. After administration, the drug selectively accumulates in abnormal tissue and is activated using light of a long wavelength (typically red light) for PDT. In addition, the photosensitizer can be excited for fluorescence visualization of the tumor [1–2]. The use of photosensitizers thus presents a convenient theranostic approach for fluorescence-guided treatment of tumors in a clinical setting [3–5].

Activation of the photosensitizer with light of a specific wavelength leads to the generation of cytotoxic reactive oxygen species, resulting in eradication of tumor cells. The main mechanisms of PDT action are direct tumor cell killing, damage to tumor vasculature and stimulation of the body's anti-tumor immune response [6–9]. PDT offers several advantages over conventional cancer treatment modalities, notably the potential to activate the body's anti-tumor immune response, even against untreated tumors [6–10]. Additionally, PDT is a localized treatment and thus does not lead to systemic toxicity. It can therefore be safely repeated with little or manageable side effects to achieve tumor control and it can be administered in combination with other treatment modalities [6].

### Tumor response monitoring

Since PDT works via the interaction of a drug, light and tissue oxygen, the outcome depends on the treatment parameters used [11–13]. Response may also vary from individual to individual even under the same treatment regime. This could be due to factors relating to the tumor microenvironment, including antitumor immunity, tumor vascularization and PDT-triggered cell survival mechanisms [14–16]. As PDT is being developed for clinical applications, there is a need to develop complementary techniques to evaluate treatment response so that the treatment regime can be personalized for best outcome. Tumor response to PDT is often assessed by observation, tumor size measurement and histopathological examination of biopsy samples. There might be a delay between assessment and decision for further treatment. Therefore techniques that can provide early response assessment in the clinic are of particular interest.

A spectrum of approaches has been reported for monitoring tumor response to PDT. These include the use of magnetic resonance imaging and its variations [17–20], positron emission tomography using various agents [21–22] and optical techniques, which have the advantages of being non-invasive and non-ionizing [23–35]. These include fluorescence microendoscopy [23], laser speckle imaging [24], photoacoustic imaging [25], optical coherence tomography [26–27] and variants of optical spectroscopy [28–35].

### Optical spectroscopy in PDT monitoring

Tissue oxygen is an essential component of the photodynamic action and shutdown of tumor vasculature is one of the major mechanisms of PDT. Measurement of tumor oxygen levels and blood flow may therefore provide insight into the tumor response to PDT. Diffuse optical spectroscopy (DOS) can be used to probe biological tissue for its optical properties such as absorption and scattering coefficients. DOS has been used to measure the tissue oxygenated hemoglobin (HbO_2_), deoxygenated hemoglobin (Hb), total hemoglobin concentration (THC), and oxygen saturation (StO_2_) levels of PDT-treated tumors [28–30, 32]. Diffuse correlation spectroscopy (DCS) allows us to measure the relative blood flow (rBF) in a tumor by using the autocorrelation function of fluctuating light intensities to calculate the average flow rate of scattering particles [28–29, 33]. This allows us to assess both the extent and time evolution of vascular damage caused by PDT. Yu et al. has previously reported the use of diffuse correlation spectroscopy, power Doppler ultrasound and broadband diffuse reflectance spectroscopy to measure tumor blood flow to provide early assessment of treatment efficacy [28]. Adapting from this approach, we developed a combined DOS and DCS system to measure changes in the tumor oxygenation level and blood flow in PDT-treated tumors [36–37]. In this study, we assessed the potential for the tumor StO_2_ and rBF levels as early indicators of treatment response to chlorin-6 (Ce6)-mediated PDT administered singly and with repeat PDT. We also monitored the long term variation of the StO_2_ and rBF levels over an extended period of time up to two weeks after treatment to study the recovery of these hemodynamic parameters to baseline values.

### Laser confocal endomicroscopy

Laser confocal endomicroscopy (LCE) is a technique that enables *in vivo* fluorescence imaging of surface and subsurface structures at the microscopic level [38]. This technique had been successfully used for *in vivo* visualization of blood vessels and blood flow [39]. Briefly, a fluorescent dye is injected into the circulation to act as a contrast agent. A handheld probe is then used to capture fluorescence images of tissue structures with microscopic resolution. We have previously reported the use of a confocal endomicroscope to study the antiangiogenic effects of PDT in combination with bevacizumab [40]. LCE was used for visualization of tumor blood vessels following treatment with PDT, bevacizumab, or PDT and bevacizumab combination therapy. In this study, LCE was used to provide information on the blood vessel architecture following Ce6-PDT.

**Figure 1:**
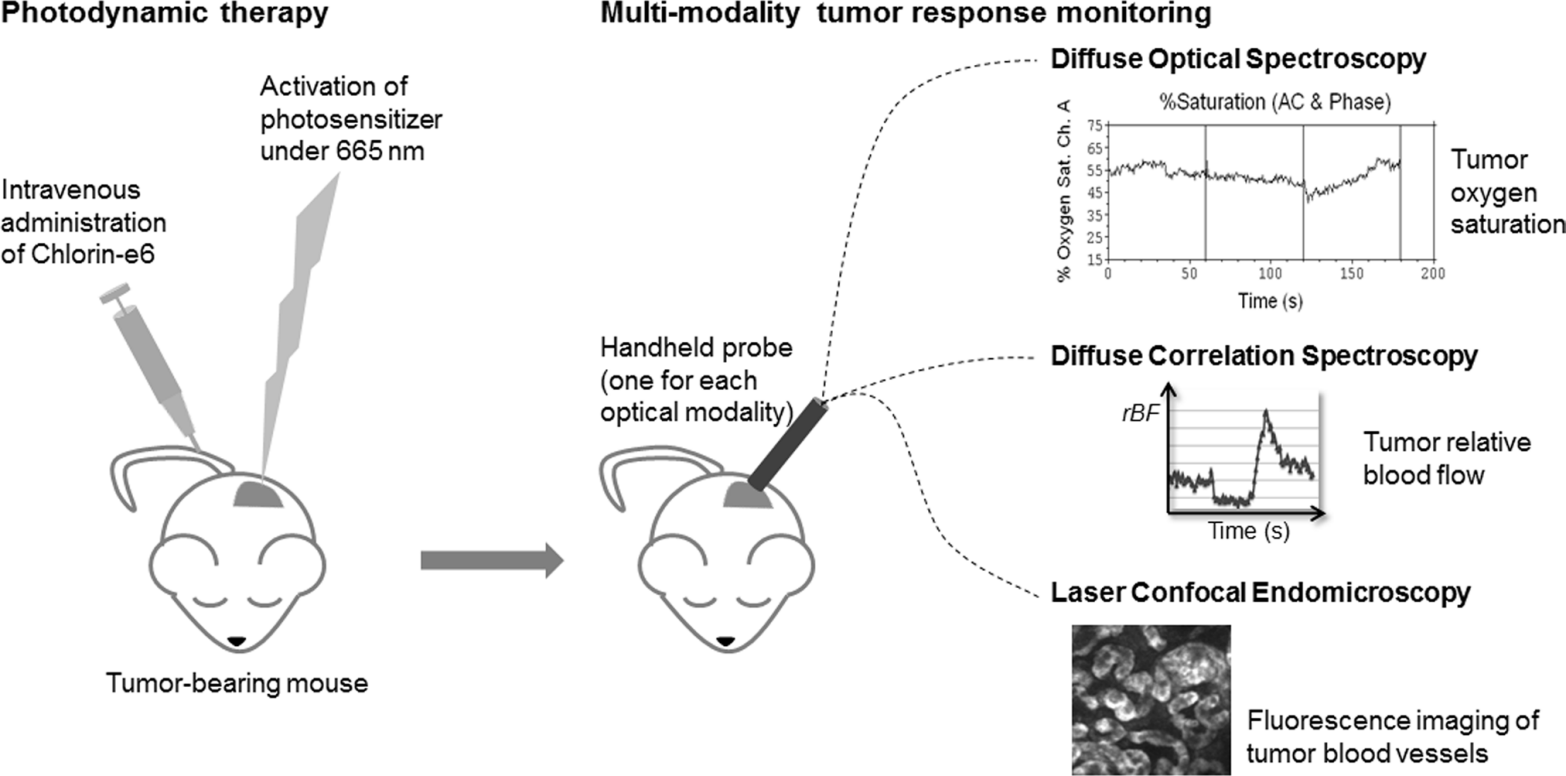
Schematic summary of the multi-modality approach used to monitor tumor response following Ce6-mediated PDT

Figure 1 shows a schematic summary of the multi-modality approach which we used to monitor tumor response following Ce6-mediated PDT. Optical spectroscopy techniques provided information on the macroscopic changes in hemodynamic parameters while fluorescence endomicroscopic imaging provided complementary information on changes to the blood vessel architecture after PDT.

## RESULTS

**Figure 2:**
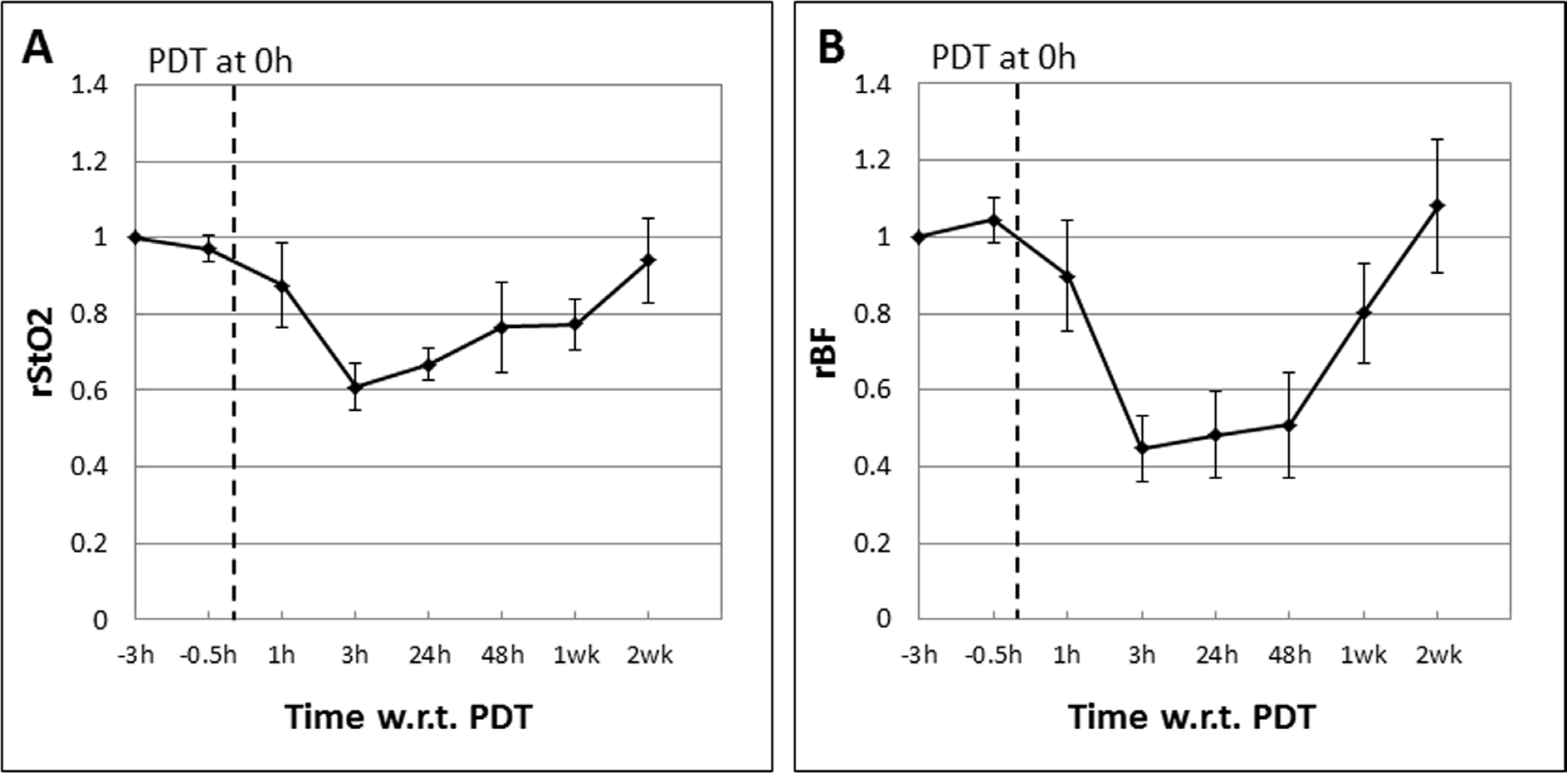
Mean tumor rStO2 (**A**) and rBF (**B**) values in complete responders (CRs; *n* = 5) expressed as ratios of the baseline readings measured before Ce6 administration at “−3 h”, 3 h prior to PDT at “0 h”. The standard errors of the mean are presented as error bars. PDT-induced decreases in both the mean rStO2 (−40%) and rBF levels (−60%) were observed at 3 h post-PDT. The mean rBF readings in these mice remained low up till 48 h post PDT. Recovery of both parameters to baseline values was observed 2 weeks after PDT.

Mouse models bearing syngeneic tumors were subjected to Ce6-PDT as described and treatment response was assessed using DOS and DCS to measure the tumor StO_2_ and rBF. Figure 2 shows the mean tumor relative StO_2_ (rStO_2_) and rBF values in complete responders (CRs; *n* = 5) expressed as ratios of the baseline readings measured before Ce6 administration at “−3 h”, 3 h prior to PDT at “0 h”. Mice in the CR group showed PDT-induced decreases in both the mean rStO_2_ (−40%) and rBF (−60%) levels at 3 h post-PDT. The mean rBF readings in these mice remained low up till 48h post PDT. Recovery of both parameters to baseline values was observed 2 weeks after PDT.

**Figure 3:**
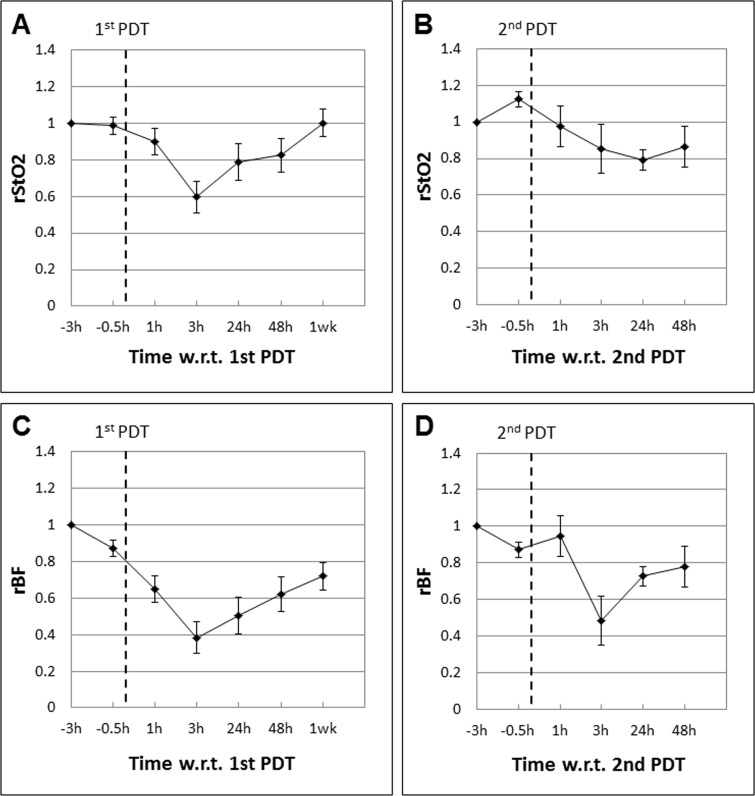
Mean tumor rStO_2_ and rBF values in partial responders (PRs; *n* = 4) expressed as ratios of the baseline readings measured before Ce6 administration at “−3 h”, 3 h prior to the first PDT (**A** and **C**) and second PDT (**B** and **D**) respectively. The standard errors of the mean are presented as error bars. The trends observed after the first and second PDT were different. The rStO_2_ values decreased by only about 15% compared to 40% following the first PDT. Although the rBF levels decreased by about 50%, these values made a quick recovery to almost 80% of baseline values within 48 h.

In partial responders (PRs) that received repeat PDT one week after the first, the mean rStO_2_ and rBF levels after the first and second PDT are plotted separately in Figure 3. Figure 3A and 3C show the mean rStO_2_ and rBF levels following the first PDT. Here we see that these parameters exhibit a trend similar to CRs, with the mean rStO_2_ and rBF levels decreasing by 40% and 60% respectively at 3 h post-PDT. However, the rBF showed a steady trend of recovery toward baseline values beginning at 24 h post-PDT (recovery of about +20% between 3 h and 48 h) shown in Figure 3C unlike in CRs. This observation is in agreement with blood flow changes as a result of the disruption of blood vessels by PDT. However, there could be an increase in the post treatment expression of survival molecules that led to tumor vasculature repair or angiogenesis [41], as evident in the recovery of rBF values. This will be discussed in more detail in the next section.

Mice in the PR group were given repeat PDT one week later following the same regimen and time points for spectroscopic measurements to see if repeat PDT resulted in the same pattern of response as the first PDT, particularly at the early time points that are relevant to early prediction of tumor response. The mean rStO_2_ and rBF levels following the second PDT are shown in Figure 3B and 3D. Unlike the trends observed after the first PDT, the rStO_2_ and rBF levels exhibited different trends following the second PDT. The rStO_2_ decreased by only 15% at 3 h post PDT compared to 40% after the first PDT. The rBF levels decreased by about 50% initially. However, these values recovered to almost 80% of baseline values within 48 h.

**Figure 4:**
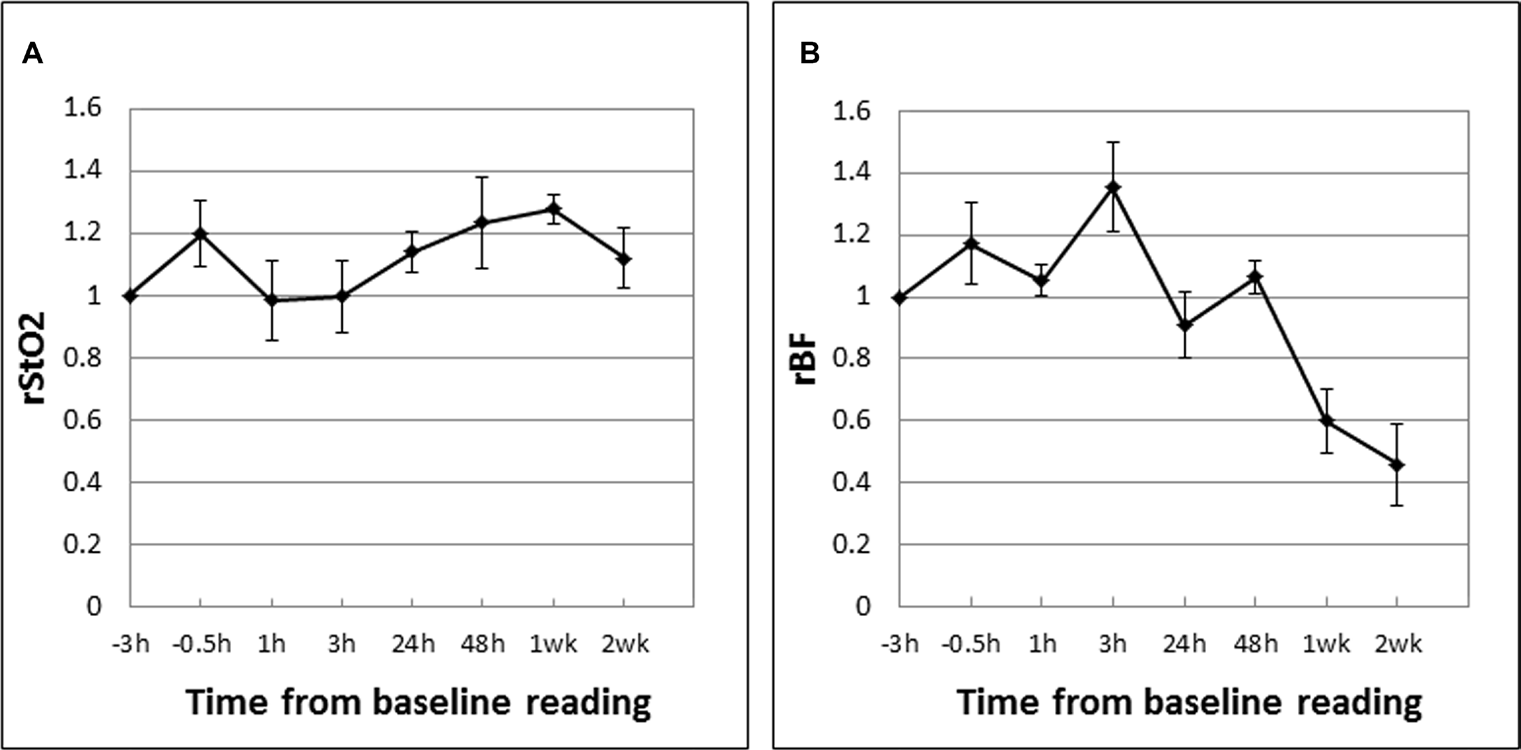
Mean tumor rStO_2_ (**A**) and rBF (**B**) values in drug-only control tumors (DC; *n* = 5) expressed as ratios of the baseline readings measured before Ce6 administration at “−3 h”. The standard errors of the mean are presented as error bars. The rStO_2_ and rBF values fluctuated above the baseline value (up to +35%) during the first 48 hours. The large decrease in rBF at 1 week (−40%) and 2 weeks (−55%) may be due to more “tortuous” tumor vasculature that restricted blood flow as the tumors grew larger (see Figure 6).

**Figure 5:**
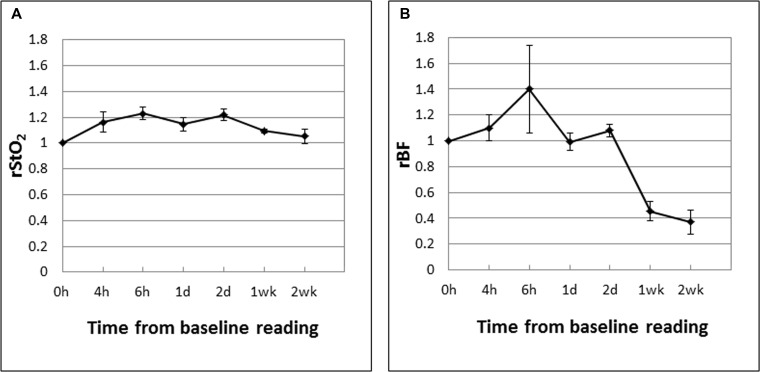
Mean tumor rStO_2_ (**A**) and rBF (**B**) values in untreated control tumors (UC; *n* = 5) expressed as ratios of the first baseline readings taken when the tumors reached the experiment size of 8 mm. The standard errors of the mean are presented as error bars. The rStO_2_ and rBF values in UCs fluctuated above the baseline value (up to +40%) during the first 48 h.

**Figure 6:**
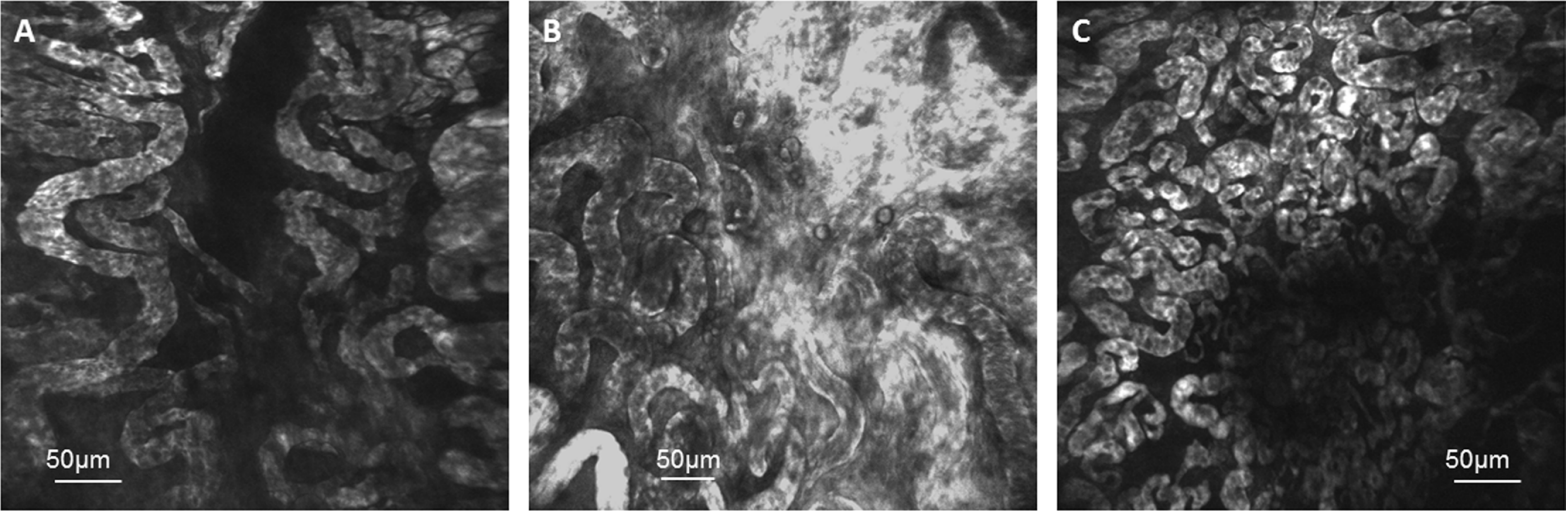
Fluorescence endomicroscopy images of tumor blood vessels from (**A**) a partial responder during a time of tumor regrowth at 3.5 weeks after reaching the experiment size of 8 mm, (**B**) a drug-only control at 2 weeks after reaching 8 mm and (**C**) an untreated control mouse at 1.5 weeks after reaching 8 mm. The “tortuous” blood vessel architecture seen in these images may explain the decrease in rBF in drug-only and untreated control tumors observed at 1 and 2 weeks when the tumors grew larger (Figures 4 and 5).

The mean tumor rStO_2_ and rBF values in drug-only control tumors (DC; *n* = 5) are shown in Figure 4. These are expressed as ratios of the baseline readings measured before Ce6 administration at “-3 h” following the same experiment and measurement time points as in the PDT groups except that there was no laser irradiation. In contrast to PDT-induced decreases in the rStO_2_ and rBF values in CRs, these parameters in DCs fluctuated above the baseline value (up to +35%) during the first 48 hours. The large decreases in rBF at 1 week (−40%) and 2 weeks (−55%) may be due to more “tortuous” tumor vasculature in that restricted blood flow as the tumors grew larger (see Figure 6). Figure 5 shows the mean tumor rStO_2_ and rBF values in untreated control tumors (UC; *n* = 5) expressed as ratios of the first baseline readings. Similar to the changes in DCs, the rStO_2_ and rBF values in UCs fluctuated above the baseline value (up to +40%) during the first 48 h. The trends of changes in DCs and UCs are thus similar to each other but different from those in CRs and PRs. This indicates that the effect of the drug by itself cannot explain the changes in blood oxygenation and flow that was observed in CRs and PRs and therefore the changes observed in Figures 4 and 5 can be attributed to the anti-vascular action of PDT.

Fluorescence images of tumor blood vessels were obtained using laser confocal endomicroscopy (Figure 6). The images were obtained from (A) a partial responder during a time of tumor regrowth at 3.5 weeks after reaching the experiment size of 8 mm, (B) a drug-only control at 2 weeks after reaching 8 mm and (C) an untreated control mouse at 1.5 weeks after reaching 8 mm. The “tortuous” blood vessel architecture observed in these images may explain the decrease in rBF in drug-only and untreated control tumors observed at 1 and 2 weeks when the tumors grew larger (Figures 4 and 5).

**Figure 7:**
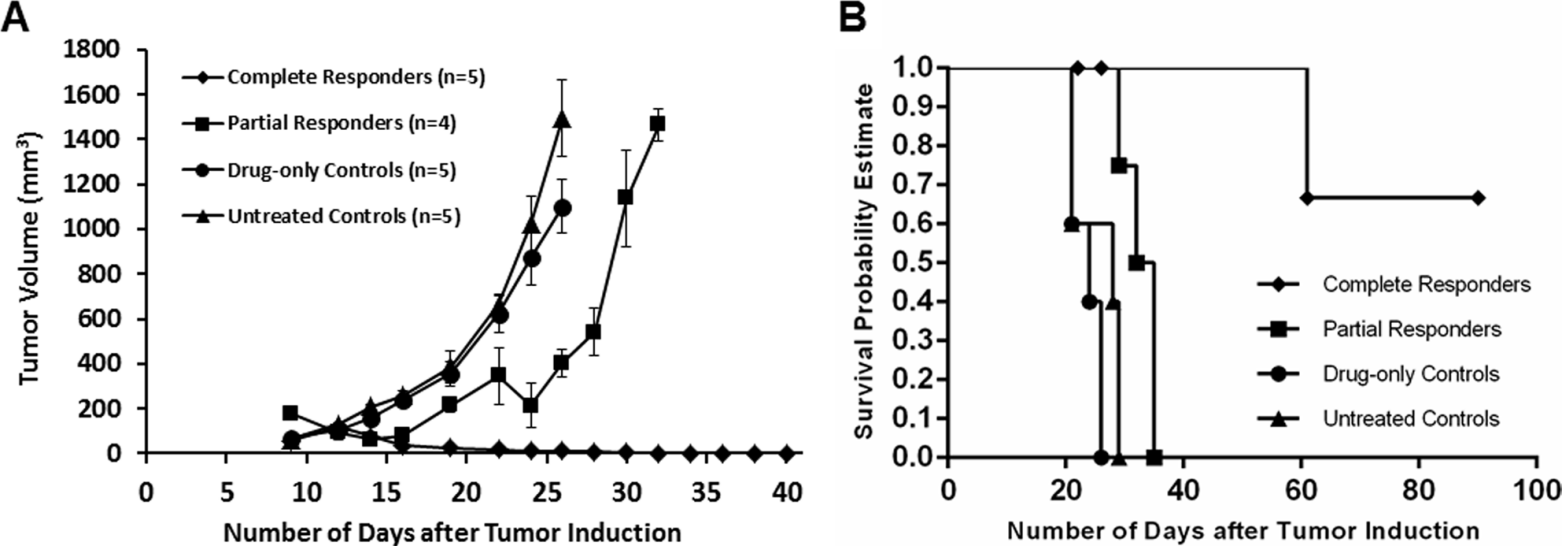
Mean tumor volume chart (**A**) and survival curve (**B**) plotted as a function of days after tumor induction show that complete responders remained tumor free for the duration of the study (up till 9 months post-PDT, data plotted till 40 days), while partial responders (mice which received repeat PDT) had a slight delay (about one week) in tumor growth progression compared to drug-only and untreated controls.

**Figure 8:**
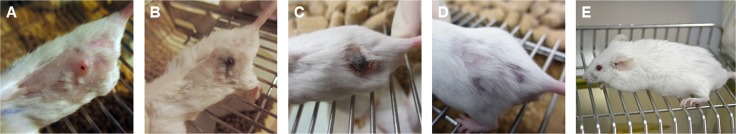
Images of a mouse from the complete responder group showing the tumor (**A**) before PDT, and at (**B**) 48 hours, (**C**) 2 weeks, (**D**) 1 month and (**E**) 6 months post-PDT. The tumor was eradicated and healing of the treatment area is seen by one month post-PDT. Long-term follow up subsequently showed no relapse of the tumor up to 9 months post-PDT.

Mean tumor volume charts plotted as a function of days after tumor induction (Figure 7) show that complete responders remained tumor free for the duration of the study and up to 6 months after PDT as shown in Figure 8E). Partial responders had a slight delay in tumor growth progression (about one week) compared to drug-only and untreated controls but eventually succumbed to tumor regrowth. Figure 8 shows images of a mouse from the complete responder group showing the tumor (A) before PDT, and at (B) 48 hours, (C) 2 weeks, (D) 1 month and (E) 6 months post-PDT. The tumor was eradicated and healing of the treatment area is seen by one month post-PDT. Long-term follow up subsequently showed no relapse of the tumor up to 9 months post-PDT.

## DISCUSSION AND CONCLUSIONS

Treatment outcome following PDT depends on the parameters used. A successful PDT outcome depends on numerous factors including the type of photosensitizer and its dose [42], the drug-light interval [43], and the total fluence and fluence rate of treatment [44]. Response may also vary from individual to individual even under the same treatment regime for various possible reasons including the following. The tumor microenvironment could play a major role in the PDT outcome. The vascular events during PDT includes endothelial cell rounding, basement membrane exposure and subsequent occlusive platelet aggregation [45] and these may affect the oxygen saturation and blood flow within the tumor microenvironment. Numerous studies have suggested that PDT-mediated vascular damage significantly contributes to long-term tumor response [46–49].

A non-invasive tumor response monitoring system is needed to understand such vascular changes and to optimize and personalize the treatment protocol. Ultrasound, optical coherence tomography (OCT), and magnetic resonance imaging have been used to monitor PDT responses [50–52]. However ultrasound suffers from various artifacts, such as acoustic shadowing and reverberation. In OCT, the resolution exceeds 10 μm, but cross sectional images can only be obtained at limited penetration depths [39]. Although MRI provides good contrast resolution, the trade-off between scan time and spatial resolution makes MRI a less popular choice. Therefore, there is a need to optimize and develop different monitoring systems for PDT.

In this study, we used a combined DOS and DCS system to assess tumor response to PDT by measuring the tumor oxygen saturation levels and blood flow (rStO_2_ and rBF) relative to pre-treatment baseline in individual tumors. The rStO_2_ and rBF levels exhibited distinctly different patterns of treatment-induced variations within the first 48 hours post-PDT between complete responders (CRs; mice with complete tumor eradication) versus partial responders (PRs; mice with initial response but suffered eventual regrowth). A large PDT-induced decrease in the rStO_2_ (40%) and rBF (60%) at 3 h post-PDT was observed in both the CRs and PRs. However, a sustained decrease in the rBF up till 48 h was observed in CRs whereas the rBF in PRs turned toward baseline value earlier at 24 h. The mice in the PR group were given repeat PDT one week later. The trends observed following the second PDT were unlike those observed after the first. The rStO_2_ showed smaller drops at 3 h post PDT compared to the larger decrease after the first PDT. Although the rBF levels decreased by about 50%, these values made a quick recovery to almost 80% of baseline values within 48 h, a level of recovery that was only observed at 1 week in both CRs and following the first PDT in PRs. It will be interesting to investigate possible reasons behind this observation in future studies.

The sustained decrease in the rStO_2_ and rBF in CRs may indicate more severe PDT-induced vascular damage, which in turn led to the better outcome observed in CRs. As post-PDT angiogenesis is known to be associated with tumor regrowth, a strong anti-vascular response may lead to complete tumor eradication. It is possible that a strong anti-vascular response is measurable. The decreased rStO_2_ and rBF levels that we measured in the first 48 h following PDT in CRs may be due to this strong response that led to complete tumor eradication in this group. In a previous study it has been shown that illumination of rat C6 glioma xenografts shortly after intravenous injection of TOOKAD induces tumor vascular damage that led to vessel constriction, hypoxia and tumor eradication [53]. Similarly we have shown that hypericin-PDT at 0.5 h drug light interval caused extensive vascular damage and these results were verified by CD31 staining whereby congested nonfunctional vessels were observed. In addition, a short drug-light interval PDT also improved tumor responsiveness in a bladder tumor xenograft model [41].

On the other hand, an earlier return of rStO_2_ and rBF toward baseline values around 24 h following the first PDT may indicate a weaker anti-vascular response in PRs. The weaker response could be the reason for tumor regrowth observed in this group. This is consistent with our previous study in which we reported recruitment of angiogenesis factors as early as 24 h post PDT [41]. It was further observed that the rBF returned to 80% of baseline values within 48 hours after PDT. This is much earlier than what was observed in both CRs and after the first PDT in PRs. The early return possibly indicates an even weaker anti-vascular response following the second PDT. The mice in this group suffered eventual tumor regrowth. Although it is beyond the scope of the current study to investigate why there was a differential response following the first and second PDT in this group of mice, we can infer, in general, that measurement of the rBF following PDT can give a good indication of the strength of the anti-vascular response and therefore early prediction of the tumor response within the first 48 hours. Our results are also consistent with an earlier study in which it was reported that mice treated with low-fluence-rate PDT showed statistically lower post-PDT microvessel density than the control and high fluence rate groups. This indicates that low fluence-rate-PDT led to a more complete vascular shutdown, while high fluence rate PDT led to an early temporary reduction of blood flow followed by a partial or possibly a complete recovery [54].

**Figure 9:**
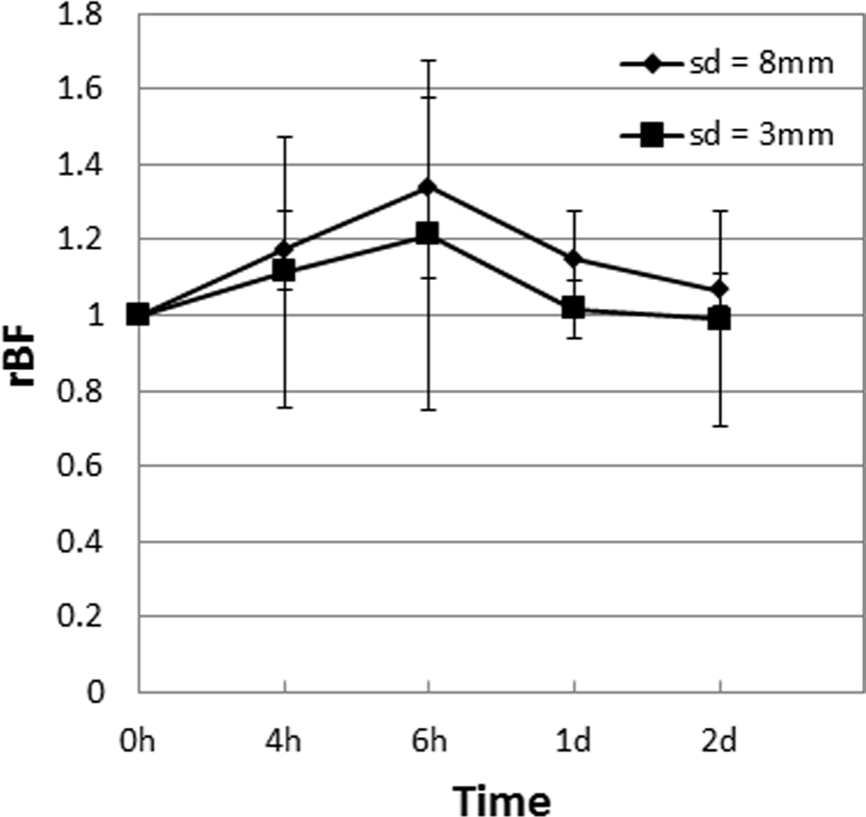
Mean tumor rBF values in muscle tissue of control mice (*n* = 4) expressed as ratios of the first baseline readings measured using a probe with a source-detector (sd) distance of 8 mm compared to one with 3 mm are shown The standard errors of the mean are presented as error bars. The measurements over a 2 day period showed that the blood flow readings are similar within a 5 mm difference in the sd distance.

In the current DOS and DCS setup, the different source-detector (sd) distances in the DOS (0.5 mm to 4 mm) and DCS (8 mm) probes results in different probing volumes. In order to get a better matching probing volume for both DOS and DCS, we are further developing a DCS probe with an sd distance of 3 mm. Preliminary measurements on mouse muscle tissue showed similar fluctuations (within 20%) in the rBF measured by the 3 mm probe compared to the readings from the 8 mm probe over a 2 day period (Figure 9). These results demonstrate that the blood flow readings are similar within a 5 mm difference in the sd distance. As DCS averages out the hemodynamics over the probing volume, the information obtained with a shorter sd distance is preserved in a longer sd.

Fluorescence endomicroscopic imaging of tumor blood vessels provided complementary information to spectroscopic measurements. The images of the tumor blood vessels were captured when the tumor has either regrown (albeit following initial growth delay after PDT) or grown unchecked in the untreated mouse and mouse that received drug only. The blood vessels in these “end-stage” tumors were observed to be large and “tortuous”. The tortuosity of these large vessels could explain the 60–70% decrease in the rBF observed at 2 weeks after the start of spectroscopic measurements in DCs and UCs. Blood flow through these untreated tumors could have been sluggish and intermittent due to the disorganized and chaotic mature of the vasculature [55]. It has also been reported that fluctuations in blood flow is related to the vessel diameter, predicting that vasoconstriction of a small vessel would increase flow resistance to a greater magnitude than the same vasoconstrictive insult in larger vessels [56]. The underlying cause of cyclic blood flow or hypoxia can be attributed to several factors, including the local hemodynamics of blood flow through tortuous tumor vasculature and vascular intussusception from rapid vessel remodeling [57].

Overall, the results from this study show that long-term tumor response to PDT can be predicted by assessing variations in the tumor rStO_2_ and rBF levels as early as 3 to 48 h post-PDT. In particular, a sustained decrease in the rBF up till 48 h post-PDT was associated with complete tumor eradication whereas an earlier return of this parameter toward baseline at 24 to 48 hours after PDT was associated with eventual tumor regrowth. By comparison, using gross volume measurement alone, an indication of tumor growth or regrowth is usually only apparent at one week post-treatment. Our future plan is to further develop a non-contact system with real-time data analysis capabilities so as to better facilitate same-day treatment response assessment in a clinical setting. This will allow us to carry out early assessment of tumor response to PDT in a clinical setting as an aid to optimization and personalization of treatment planning.

## MATERIALS AND METHODS

### *In vivo* photodynamic therapy

**Figure 10:**
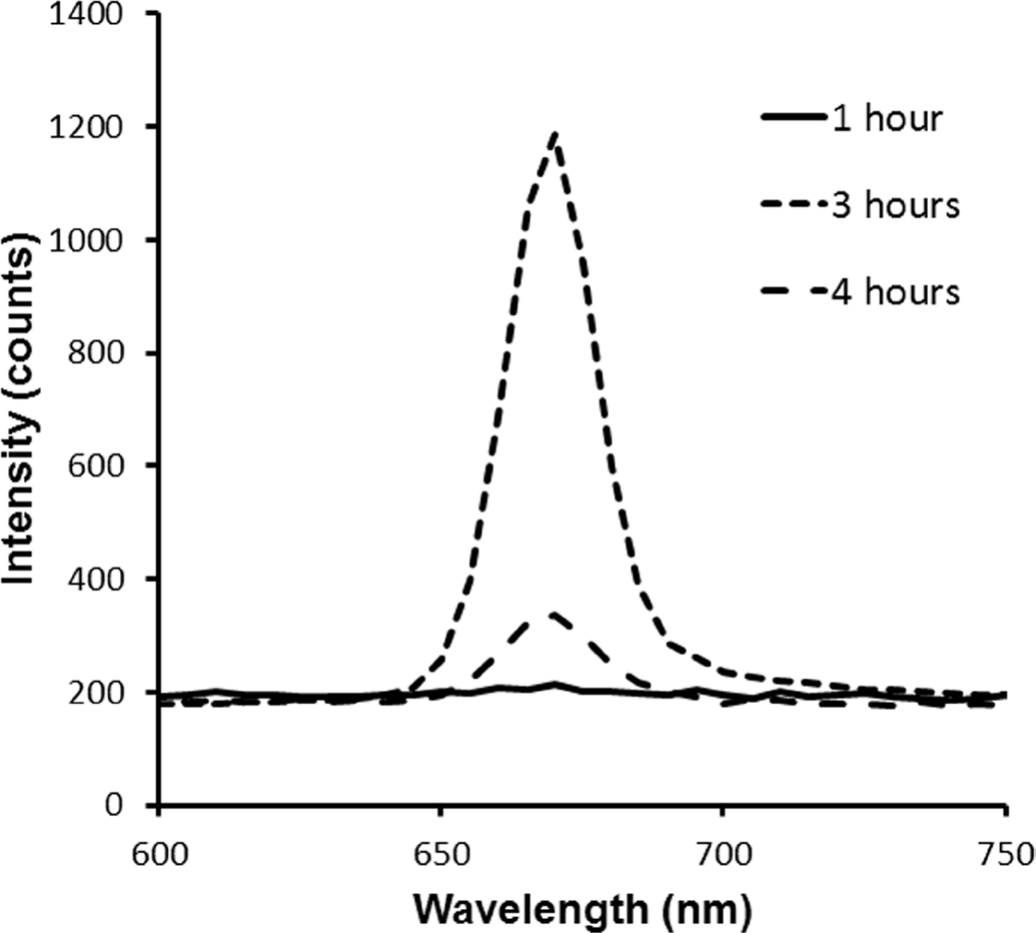
Fluorescence intensities measured *in vivo* in tumors using a spectrometer at 1 h, 3 h and 4 h after intravenous administration of Ce6

Sub-cutaneous syngeneic tumors of mouse mammary carcinoma (EMT-6) were induced in Balb/c mice (InVivos, Singapore). Tumors were subject to PDT when the tumors reached approximately 8 mm in diameter. The photosensitizer chlorin-e6 (Ce6) (ApoCare GmbH, Germany) was administered intravenously at a dose of 5–10 mg/kg. At 3 h post drug administration, light at 665 nm was delivered by a laser (Biolitec, Germany). The laser beam was expanded to illuminate a diameter of 2.5 cm on the treatment surface. The light was delivered via an optical fiber at 85–130 mW/cm^2^ for a light dose of 100–200 J/cm^2^. Mice were anesthetized with isoflurane during PDT as well as during DOS, DCS and LCE measurements to minimize mouse movement. The 3 h drug-light interval was determined by quantitatively measuring the fluorescence intensity using a spectrometer at 1 h, 3 h and 4 h after administration of the photosensitizer (Figure 10). At 3 h post drug administration, the fluorescence intensity was observed to be the highest.

The oxygen saturation and blood flow readings were calculated as ratios relative to the baseline values in each mouse before Ce6 administration (abbreviated as rStO_2_ and rBF here). The tumor rStO_2_ and rBF were measured up to 2 weeks post-PDT. Tumor sizes were measured every 2 days. Complete responders (CR; *n* = 5) were mice with tumors that regressed following PDT and remained tumor free. Partial responders (PR; *n* = 6) were mice with tumors that partially regressed following PDT but regrew. These mice were given repeat PDT one week later to see if repeat PDT resulted in the same pattern of response as the first PDT. The rStO_2_ and rBF readings that were measured following the second PDT were calculate as ratios relative to baseline values recorded before the second dose of Ce6. The rStO_2_ and rBF values were similarly measured relative to pre-Ce6 baselines in drug-only control tumors that received 10 mg/kg Ce6 but no light (DC; *n* = 5) . In untreated control tumors (UC; *n* = 5), rStO_2_ and rBF were calculated relative to the first set of baseline values measured. The study was approved by the Institutional Animal Care and Use Committee (IACUC) of the Singapore Health Services.

### Diffuse optical spectroscopy (DOS) setup

For DOS, a frequency domain tissue oximeter (OxiplexTS, ISS Inc., USA) with a customized optical probe was used to deliver intensity-modulated radio-frequency light to the tumor at 690 nm and 830 nm. Four source-detector distances were used for each wavelength. The optical probe consisted of a fiber bundle with 8 source fibers and 1 detector fiber. The tips of the source and detector fiber bundles in the probe are aligned. The source to detector (sd) distance varied between 0.5 mm to 4 mm. Water concentration was assumed to be 75%. The tumor HbO_2_, Hb, THC, and rBF, as well as the μ_α_ and μ_s_’ which are the absorption and reduced scattering coefficients of the tumor tissue respectively were recorded. Those measurements were updated at a 2 Hz rate, and the mean value, together with the standard deviation, was calculated over a 1 minute measurement interval.

### Diffuse correlation spectroscopy (DCS) setup

For DCS, a continuous-wave, long coherence length (> 10 m) 785 nm laser (CrystaLaser, USA) was coupled into a multi-mode optical fiber with a 400 μm core diameter to illuminate scattering particles in tumor tissue. A single-mode fiber gathers photons 8 mm away from a single speckle emitted from the tumor surface. Light intensity fluctuations were detected by a photon counting avalanche photodiode (APD; Perkin-Elmer, Canada). The output of the APD was a stream of transistor-transistor logic pulses. These pulses were fed to a 32-bit, eight input channels counter/timer board through a shielded input-output connector block for data acquisition devices (SCB-68; National Instruments, USA) to be counted. The absorption and reduced scattering properties, μ_α_ and μ_s_’ of the tumor tissue measured by DOS at 830 nm were used as fixed parameters. The blood flow index is defined as BFI = αD_B_, where *α* is the volume fraction of moving scatterers out of all scatterers, and D_B_ is the effective diffusion coefficient of scatterers. The data was fitted using a Brownian motion model and the relative blood flow, rBF, was calculated from the ratio BFI / BFI_baseline_. The rBF value was updated every second in real time, and the mean value and standard deviation was calculated over a 1 minute measurement interval.

### Laser confocal endomicroscopy (LCE) of tumor blood vessels

Laser confocal endomicroscopy (LCE) was performed on tumors between 1.5 to 3.5 weeks after reaching the experiment size of 8 mm. To prepare mice for LCE, a solution of fluorescein isothiocyanate (FITC)-labeled dextran with molecular weight 150 kDa (Sigma Aldrich, USA) was prepared at a concentration of 25 mg/ml. The FITC dextran was administered intravenously at a dose of 10 μl solution per gram weight of the animal. The mice were then anesthetized with isoflurane. The skin overlaying the tumor was carefully removed to expose the tumor. *In vivo* fluorescence microscopic imaging of tumor blood vessels was carried out approximately 15 to 30 min later. Fluorescence LCE was carried out using the Optiscan FIVE 1 system (Optiscan Pty Ltd., Australia). A 488-nm excitation laser was coupled into a single optical fiber that acted as both a point source and a point detection pinhole for confocal imaging within a field of view of 475 μm × 475 μm. The system is capable of imaging of tissue with a lateral resolution of 0.7 μm and an axial resolution of 7 μm. The miniaturized components of the x-y scanning and z axis actuator were housed within a handheld rigid probe (model RBK6315A) with a probe shaft of length 150 mm and diameter 6.3 mm. Fluorescence images could be captured at various imaging depths along the z axis ranging from the surface to a subsurface depth of about 250 μm. The laser power output at the distal tip of the probe was adjusted between 700 to 1000 μW for best contrast. Fluorescence signals were collected through a 505–750 nm emission filter.
