# Correction: Early assessment of tumor response to photodynamic therapy using combined diffuse optical and diffuse correlation spectroscopy to predict treatment outcome

**DOI:** 10.18632/oncotarget.26742

**Published:** 2019-02-22

**Authors:** Patricia S.P. Thong, Kijoon Lee, Hui-Jin Toh, Jing Dong, Chuan-Sia Tee, Kar-Perng Low, Pui-Haan Chang, Ramaswamy Bhuvaneswari, Ngian-Chye Tan, Khee-Chee Soo

**Affiliations:** ^1^ Division of Medical Sciences, National Cancer Centre, Singapore; ^2^ Division of Bioengineering, School of Chemical and Biomedical Engineering, Nanyang Technological University, Singapore; ^3^ Division of Surgical Oncology, National Cancer Centre, Singapore; ^4^ Nanyang Technological University, Singapore; ^5^ Current address: Harvard Medical School and Wellman Center for Photomedicine, Massachusetts General Hospital, Boston, MA, USA; ^6^ Current address: Daegu Gyeongbuk Institute of Science and Technology, Daegu, Korea

**This article has been corrected:** The correct author name is given below:

**Patricia S.P. Thong**

Original article: Oncotarget. 2017; 8:19902-19913. https://doi.org/10.18632/oncotarget.15720

